# Assessing the Degeneration of Cassava Under High-Virus Inoculum Conditions in Coastal Tanzania

**DOI:** 10.1094/PDIS-05-18-0750-RE

**Published:** 2019-07-19

**Authors:** Rudolph R. Shirima, Daniel G. Maeda, Edward E. Kanju, Silver Tumwegamire, Gloria Ceasar, Edda Mushi, Caroline Sichalwe, Kiddo Mtunda, Geoffrey Mkamilo, James P. Legg

**Affiliations:** 1 International Institute of Tropical Agriculture (IITA), Dar es Salaam, Tanzania; 2 University of Dar es Salaam, Dar es Salaam, Tanzania; 3 Tanzania Agricultural Research Institute, Kibaha, Tanzania; 4 Tanzania Agricultural Research Institute, Tabora, Tanzania; 5 Tanzania Agricultural Research Institute, Dodoma, Tanzania

**Keywords:** AUDPC, AUYPC

## Abstract

Cassava brown streak disease (CBSD), caused by cassava brown streak ipomoviruses (CBSIs), has become the most debilitating biotic stress to cassava production in East and Central Africa. Lack of CBSD-resistant varieties has necessitated the search for alternative control measures. Most smallholder farmers reuse stems from previous crops for planting in the new season. Recycling planting material in this way can lead to “degeneration” owing to the compounding effects of disease. In this study, degeneration was defined as the increase in CBSD incidence and reduction in marketable root yield over time. An experiment was established to study the rates of degeneration in selected cassava varieties Chereko, KBH2002_135, Kipusa, Kizimbani, and Mkuranga1 and cultivars Kiroba and Kikombe under high-CBSD inoculum conditions in Bagamoyo, Tanzania from 2013 to 2017. The experiment was replicated across two seasons: the first planted during the long rains (Masika) between March and June and the second planted during the short rains (Vuli) between October and December. Mean abundance of the whitefly vector (*Bemisia tabaci*) was much greater during the Vuli season (>19 insects per plant) than the Masika season (<2 insects per plant). CBSD shoot symptoms occurred naturally and were observed only on Kikombe, Kiroba, and Kipusa. New materials had overall lower CBSD shoot incidences (1.5%) compared with recycled materials (6.9%) in Masika, although no significant differences were obvious in Vuli. However, Masika (8.7%) had an overall lower CBSD shoot incidence than Vuli (16.5%) in the varieties that had shoot symptoms. CBSD root incidences were higher in Vuli (10.3%) thanMasika (4.4%), and root yields in Masika (29.4 t/ha) were significantly greater than those in Vuli (22.5 t/ha). The highest percentage of roots rendered unusable owing to CBSD was observed in Vuli. There was significantly higher unusable root incidence in recycled materials (3.7%) than in new materials (1.4%) in Masika but not in Vuli. Overall root yield was similar between recycled and new materials in either season. Significant reductions in root yield over the course of the experiment were observed both in Masika and Vuli, whereas changes in marketable yield were significant only in Masika. Differences in the response of varieties to degeneration led to the identification of four degeneration patterns, namely “strong,” “moderate,” “mild,” and “delayed” degeneration. The strongest effects of degeneration were most obvious in the susceptible cultivar (Kikombe), which also had the lowest marketable yield in either season. Seasonal differences were a key driver of degeneration, because its effects were much greater in Vuli than Masika. To the best of our knowledge, this work reports the first study of degeneration caused by cassava viruses.

Cassava (*Manihot* esculenta *Crantz*) is one of the most important staple food crops in sub-Saharan Africa. Its production is affected by many biotic and abiotic factors, but cassava brown streak disease (CBSD) caused by cassava brown streak ipomoviruses (CBSIs) (Mbanzibwa et al. 2009; Winter et al. 2010) is particularly threatening, because its severity and spread have increased greatly since 2004 (Alicai et al. 2007; Ntawuruhunga and Legg 2007). CBSIs are propagated through planting infected cuttings and transmitted from plant to plant by the whitefly vector *Bemisia tabaci* (Genn.) (Maruthi et al. 2005, 2017). CBSD affects the quality and quantity of the tuberous roots, leading to economically significant annual crop losses (Hillocks et al. 2001; Ndyetabula et al. 2016). CBSD is currently localized to the cassava-growing regions of East and Central Africa. Spread to unaffected African regions can be controlled if proper management strategies are put in place (Legg et al. 2014a, b). The continuing expansion of the areas affected in Central Africa, particularly in the Democratic Republic of Congo, threatens food security in some of the most vulnerable parts of Africa, where cassava is the primary staple. The effects of the disease will be greater still if the disease spreads as far as Nigeria in West Africa, because this is currently the world’s largest cassava producer (Legg et al. 2011, 2015; Patil et al. 2015). To mitigate the foreseen food security crisis, several breeding programs have produced large numbers of “elite” varieties, which are often characterized by breeders and virologists as tolerant or sometimes resistant to CBSD (Kaweesi et al. 2014; Maruthi et al. 2014; Mohammed et al. 2015).

In Tanzania, cassava is cultivated in all regions, but the most important production areas include the northwest (around Lake Victoria), the coastal zone in the east, and all parts of southern Tanzania (FAO 2005; IITA 2015). Many cassava farmers recycle stems from the previous crop to use as planting material for the new season. However, recycling of planting material is the main source of primary inoculum in cassava plantations within the subsequent crop cycles (FAO 2006). As a vegetatively propagated crop, cassava is prone to virus diseases (Hillocks and Thresh 2000; Calvert and Thresh 2002). Many farmers, in the absence of proper training and intervention strategies, do not select clean planting material. Consequently, infections are maintained from one season to the next (Gildemacher et al. 2011; Hillocks and Jennings 2003; Legg et al. 2011). Whereas the selection of symptomless planting material has proven to be effective with cassava mosaic disease (CMD) (Fargette et al. 1988; Mallowa et al. 2011; Thresh et al. 1994), such an approach is impracticable with CBSD owing to the difficulty of recognizing foliar symptoms as well as the prevalence of asymptomatic CBSIinfected plants. This makes it difficult to use farm-based management practices, such as roguing (Legg et al. 2011).

Although the planting of clean seed would be an effective way for farmers to reduce the impact of CBSD, in most situations, the seed is not available, and where it is available, farmers may not be able to afford it. It is a general feature that many farmers cannot get quality seed every season because of the cost and availability; therefore, their main stock of material for planting is recycled seed from previous plantings (Gildemacher et al. 2011). The quality of recycled planting material gradually degenerates owing to disease, agronomic, and or other environmental factors (Gildemacher et al. 2011). “Degeneration” is the gradual decline in the quality of planting material over repeated cropping cycles. As previously done for CMD, considerable research effort has been directed toward the development of CBSD-resistant varieties. Significant progress has been made with conventional breeding approaches (Jennings 1957; Kawuki et al. 2016), and varieties with increasing levels of CBSD resistance are gradually being introduced to the seed systems of CBSD-affected countries. As newly developed varieties become available in sufficient quantities for dissemination to farmers, it will be important to understand their long-term performance under conditions of CBSD inoculum pressure. Degeneration will vary for different varieties depending on the environmental conditions and the relative resistance of the variety to CBSD. Although there are no published reports for degeneration of cassava in Africa, a study on sweet potato degeneration suggested that it was more economically effective for farmers to recycle tolerant cultivars than to source new ones every season, because there were no significant yield differences in subsequent plantings (Adikini et al. 2015). For potato, Whitehead (1930) reported that virus infection, climatic, seasonal, and varietal factors as well as the age of the seed stock influenced the rate of degeneration of certain potato varieties. For cassava farmers, it will be essential to know the length of time over which varieties will continue to provide favorable yields. For individual entrepreneurs and seed companies selling the varieties, they will need to know how seed degeneration will affect repeat sales. For example, if a variety is highly resistant, farmers may not see the need to purchase clean seed again; however, if the variety degenerates too rapidly, farmers may not see the value of the variety at all. An intermediate condition might, therefore, be most likely to favor both seed sellers and producers.

In the study reported here, we have made the first attempt to characterize degeneration of cassava varieties under high-CBSD inoculum pressure conditions. It was anticipated that this would provide a model for more extensive studies to be conducted over a wider range of environmental conditions in parts of Africa affected by CBSD. Ultimately, an improved understanding of the degeneration process will allow breeders to produce varieties that specifically target the needs of both seed and root producers.

## Materials and Methods

**Experimental site.** The degeneration experiment was conducted in a high-CBSD disease pressure site in Bagamoyo, Tanzania (latitude: −6.5542; longitude: 38.9140; altitude: 41.5 m). This is in the coastal part of eastern Tanzania ~50 km north of the city of Dar es Salaam. The rainy season in this region exhibits a bimodal rainfall pattern, with the main rainy season between March and June (Masika) and the short rainy season between October and December (Vuli) (FAO 2005; Kijazi and Reason 2009).

**Planting material.** Planting material was obtained from four elite released varieties (Chereko, Kipusa, Kizimbani, and Mkuranga1), one prerelease breeding clone (KBH2002_1350; referred to as variety for simplicity), and two check cultivars (Kiroba and Kikombe), which were maintained at a clean seed site in Tanga, northeastern Tanzania. Clean seed sites in Tanzania are managed with stringent conditions to maintain the health of the cassava plants, including bimonthly monitoring visits during which any plants with virus symptoms are rogued. In addition, leaf samples are collected for virus testing before harvesting stems. The tolerance level for CBSD in clean seed sites is <2% incidence. Kiroba is known to be tolerant to CBSD and CMD, whereas Kikombe is susceptible. Each of the other varieties was assumed to show field resistance to both CBSD and CMD. For planting year 1 of each of the Masika and Vuli seasons, virus-negative stem cuttings ~20-cm long obtained from the clean seed site in Tanga (new) and verified by testing for CBSIs using virus-specific TaqMan real-time reverse transcription polymerase chain reaction (RT-PCR) assay (Adams et al. 2013; Shirima et al. 2017) were prepared and planted in a randomized complete block design with three replications and 42 cuttings per plot. Plant spacing was 1 × 1 m. Subsequent planting cycles were planted in a similar manner with planting material (recycled) selected from the previous crop by a farmer. Five to 10 stems were selected per plot from each replication, pooled together, and cut to make the required number of cuttings. These were then randomly distributed to the three replications of the new planting cycle. On this occasion, however, a batch of new cuttings was introduced using planting material obtained from the clean seed site in Tanga. To accommodate this, the experiment was planted in a split plot design. New cuttings and recycled cuttings were planted as split plots, and varieties were main plots. Two experiments were conducted concurrently: one planted during Masika in May 2013 (Masika season) and the second in November 2014 (Vuli season). The second and third Masika plantings were planted in May 2014 and 2015, whereas the fourth planting was in May 2016. The second Vuli planting was done in November 2015, and the third was in November 2016. Plant establishment was recorded at 1 month after planting (MAP). Any gaps resulting from cuttings not sprouting were filled with cuttings of the appropriate treatment. No additional replacement of dead plants was done after this stage. In year 2 of the Vuli season, replacement planting material for the new material was not available for Chereko, KBH200_135, Kikombe, and Kizimbani, and these were, therefore, treated as missing data.

**Disease and vector abundance.** *Foliar CBSD assessments*. Plants in the net plot (20 plants per plot excluding all of the outer guard rows of each plot) were assessed for CBSD symptoms once every month starting at 1 MAP until harvest (12 MAP). The remaining 22 plants surrounding the net plot were treated as guard rows. Foliar CBSD incidence (plant shoot incidence) was calculated as the percentage of plants with CBSD leaf symptoms severity score class of$2 averaged across the monthly scores of each growing cycle. Leaf severity assessment was done using a scoring scale of 1 to 5, where 1 = asymptomatic; 2 = slight leaf feathery chlorosis without stem symptoms; 3 = pronounced leaf feathery chlorosis, mild stem lesions, and no stem dieback; 4 = severe leaf feathery chlorosis, severe stem lesions, and no dieback; and 5 = defoliation, severe stem lesions, and dieback (Gondwe et al. 2003). Mean CBSD severity score was calculated across the growing cycle using monthly average score data points. Five plants were randomly selected and tagged for leaf sample collection and subsequent virus testing, which was done monthly from 2 to 12 MAP. During leaf sampling, the central lobe was picked from the third fully expanded leaf counting from the first fully open leaf at the top of the tallest shoot. Collected leaf samples were affixed to plain newsprint paper and preserved dry before being used in the laboratory for nucleic acid extraction and virus testing.

*Vector abundance.* For each planting season, counts of *B. tabaci* whiteflies were conducted monthly from 1 to 8 MAP, after which populations had declined to low levels. Counting was done from the five topmost leaves of the tallest shoot for each of the 20 net plot plants.

**Yield and CBSD root symptoms assessment.** *CBSD root symptoms and incidence*. CBSD root symptoms were assessed for all roots of each plant in the net plot. Scoring was done by cutting each root transversely five times and observing necrotic rot on each cut. The first cut was made at the base of the root close to the point of attachment to the stem, whereas the fifth cut was made at a point near to the root tip. Root symptoms were scored on a 1 to 5 scale, where 1 = asymptomatic, 2 = mild, 3 = moderate, 4 = severe, and 5 = very severe corky dry necrotic root rots (Gondwe et al. 2003; Kaweesi et al. 2014). Mean severity scores were calculated as the averages of all of the severity score classes of$2. Several incidences of CBSD root symptoms were calculated. Plant total incidence was the percentage of plants with CBSD symptoms in either shoots or roots. Plant root incidence was the percentage of plants with one or more roots with severity score class of $2 in one or more of the five cross-sectional cuts made through the root. Root incidence was the percentage of roots with CBSD severity score class of $2. Cross-sectional root cuts incidence was the percentage of crosssectional cuts with severity score class of $2. Unusable root incidence was the percentage of roots with at least one cut with severity score of ≥3.

*Shoot and root yield, dry matter, and harvest index*. Harvesting was conducted at 11 to 13 MAP, and yield (root and shoot biomass) was recorded using the 20 net plot plants. Weights of individual fresh roots were recorded using a balance and calculated to get total root weight per plot. Shoot weight for each net plot plant was also individually measured, and the yield was calculated. Root dry matter (DM) content was determined using the specific gravity method (Teye et al. 2011) using freshly harvested healthy roots from five randomly selected plants per plot. The same plants were used for root harvest index (HI) calculation, which was the ratio of the total root weight in tons per hectare to the total biomass (sum of the total root and shoot weights in tons per hectare). HI was presented as this ratio multiplied by 100. Root marketable yield was calculated as total yield minus the yield of unusable roots. Area under the disease progress curve (AUDPC) was calculated for unusable root incidence, whereas area under the yield progress curve (AUYPC) was calculated for marketable root yield; both used equation 1 for each plot (replication) and rainy season for Kikombe, Kipusa, and Kiroba:

$$\text{AUYPC/AUDPC=}\sum_{i}^{n-1}\left[\left\{\frac{Yi+y(i+1)}{2}\right\}\text{X(t(i+1)}-\text{ti)}\right]$$ (1)

Yi = marketable yield/unusable root incidence in the ith year, ti = ith year, and n = number of years in which AUYPC was recorded. The unit for AUYPC is tons per hectare planting cycle unit (pcu), whereas for AUDPC, it is percentage pcu.

**Fig. 1:**
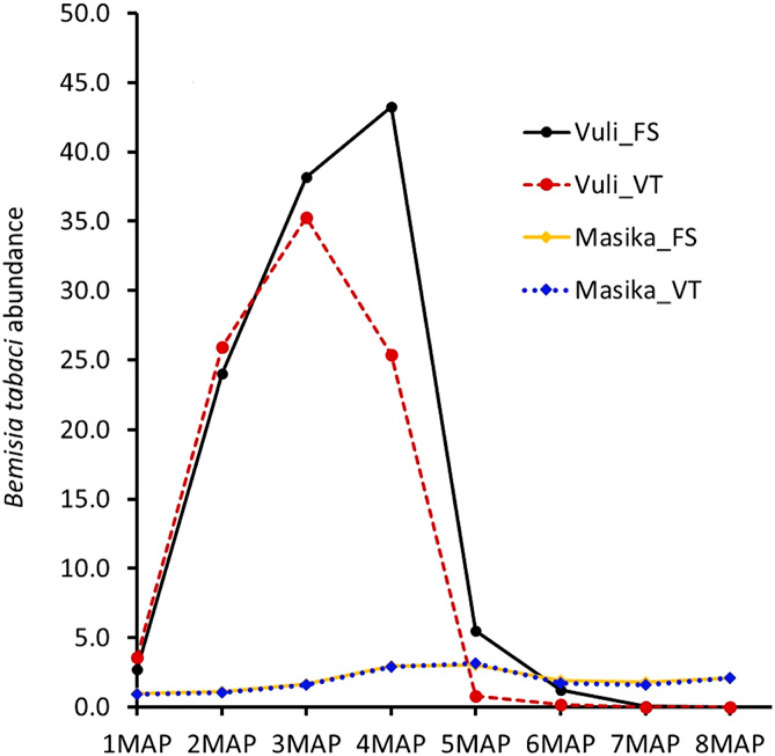
Comparison of *Bemisia tabaci* abundance recorded for selected cassava varieties planted during the Masika and Vuli seasons at Chambezi, Bagamoyo, Tanzania from 2013 to 2016. Monthly whitefly mean counts recorded from five fully expanded apical leaves of the tallest shoot of each of the net plot plants (20). For Masika, 1 to 8 month after planting (MAP) = June to January. For Vuli, 1 to 8 MAP = December to July. FS, recycled; VT, new.

**Surrounding CBSD pressure**. At 2 MAP, disease incidence and vector abundance were assessed from fields surrounding the experimental plot to determine the inoculum pressure. Fields with cassava older than the experiment were sampled within a radius of 250 m. This distance was measured by means of a GPS unit walking from a central point on the corresponding edge of the experimental plot to the center of the surrounding field. This was repeated for all fields around the four faces of the experimental plot. CBSD leaf symptom severities as well as *B. tabaci* counts were recorded for 100 plants along two diagonals (50 plants per diagonal) of each sampled surrounding field. Where the number of plants was <100 in a given field, all plants in that field were assessed. For each field, plant population was estimated, and the age of the crop was recorded. Finally, CBSD disease index (CBSD pressure) was calculated using the method of Legg et al. (1997).

**Real-time RT-PCR detection of CBSIs.** Approximately 35 mg of dried leaf tissue was ground per sample (plant) using a GenoGrinder (SPEX SamplePrep2010), and nucleic acids were extracted as described previously (Abarshi et al. 2010; Maruthi et al. 2002; Shirima et al. 2017). Nucleic acid pellets produced in this way were resuspended in 100 µl of 1× TE and treated with DNAse according to the manufacturer’s instructions (Sigma-Aldrich). The resulting RNA sample was used immediately for CBSI testing using a TaqMan assay. The remaining RNA samples were stored at −80°C for future use. *Cassava brown streak virus* (CBSV) and *Ugandan cassava brown streak virus* (UCBSV) TaqMan assays were performed separately to detect each of the two CBSI species encountered (Adams et al. 2013; Shirima et al. 2017).

**Data analysis.** CBSD incidence was calculated using the formula number of plants with CBSD symptoms over number plants assessed × 100. Mean CBSD severity was calculated as the average of score classes 2 to 5 observed per plot (variety). *B. tabaci* mean abundance and CBSD mean severity data were subjected to log10 transformations before statistical analysis, whereas CBSD incidence data were subjected to arcsine square root transformation. Vector abundance, CBSD incidence, severity, shoot and root yields, AUDPC, and AUYPC were analyzed using the generalized linear model, whereas means were separated using the Student–Newman– Keuls test using the Statistical Analysis System (SAS; SAS Institute Inc., version 9.4). Pearson correlation coefficients were used to evaluate relationships between CBSD shoot and root incidences and severity and shoot and root yields, whereas associations between shoot and root severity values with the different CBSD incidence types were analyzed using linear regression in SAS. Interactions among the different factors (year, season, planting material, and variety) affecting CBSD and yield changes over time were resolved using generalized linear mixed models (Proc GLIMMIX; SAS) by fitting replication as the random term, whereas year, variety, season, and planting material were the fixed terms, and least squares means were compared using Tukey’s multiple comparison test.

## Results

**Vector abundance.** Year (*P* = 0.006) and season (*P* = 0.005) effects were significant for vector abundance. *B. tabaci* was significantly more abundant in the Vuli than the Masika season (*P* < 0.0001) (Fig. 1), whereas differences among new and recycled planting materials were not significant. The overall mean whitefly abundance of 19.6 insects per plant recorded in Vuli is considered high (Legg et al. 2011) as opposed to the low abundance (1.9 insects per plant) recorded in Masika. There were significant differences (*P* < 0.0001) in *B. tabaci* abundance on the different varieties: Kipusa had the highest (3.7 [Masika] and 35.4 [Vuli]) (Table 1) mean whitefly numbers per plant, and Mkuranga1 had the lowest (0.7 [Masika] and 9.1 [Vuli]). There was no large change in whitefly numbers over the course of the Masika season (Fig. 1) compared with the Vuli, where there was a sharp increase followed by a decline (Fig. 1). A similar general trend was observed for changes in whitefly abundance over time within each of the two seasons, with peaks around 3 to 4 MAP. Rainy season × year effects on whitefly abundance were significant (*P* < 0.0001). However, the interactions variety × season × planting material and between season and planting material were not significant for whitefly abundance.

**Table 1:** Distribution of Bemisia tabaci and cassava brown streak disease shoot incidences of cassava varieties planted during Masika and Vuli seasons at Cham-bezi, Bagamoyo, Tanzania from 2013 to 2017^z^

Year				1			2				3				4	
Season	Masika	Vuli	Overall	Masika	Vuli		Masika		Vuli		Masika		Vuli		Masika	
Selection	*B. tabaci abundance*			VT	VT		FS	VT	FS	VT	FS	VT	FS	VT	FS	VT
*N*	21	15	36	Plant shoot incidence												
Chereko	1.5 b	15.4 bc	8.5 a	0.0 a		0.0 a	0.0 a	0.0 b	0.0 a	–	0.0 a	0.0 a	0.0 a	0.0 a	0.0 a	0.2 a
KBH2002_135	1.8 b	17.2 bc	9.5 a	0.0 a		0.0 a	0.0 a	0.0 b	0.0 a	–	0.0 a	0.0 a	0.0 a	0.0 a	0.4 a	0.0 a
Cultivar Kikombe	2.1 b	16.2 bc	9.2 a	0.6 a		16.6 a	5.2 a	0.2 b	34.5 a	12.5 a	17.9 a	3.7 a	45.6 a	43.7 a	42.3 a	1.3 a
Kipusa	3.7 a	35.4 b	19.6 a	0.2 a		14.7 a	0.2 a	0.0 b	10.2 a	0.0 a	5.6 a	0.0 a	1.0 a	0.6 a	0.6 a	0.8 a
Cultivar Kiroba	1.8 b	17.1 bc	9.5 a	1.8 a		3.2 a	21.5 a	21.2 a	17.4 a	2.9 a	21.2 a	5.4 a	31.6 a	23.7 a	30.8 a	2.6 a
Kizimbani	1.7 b	23.9 b	12.8 a	0.0 a		0.0 a	0.0 a	0.0 b	0.0 a	–	0.0 a	0.0 a	0.0 a	0.0 a	0.2 a	0.4 a
Mkuranga1	0.7 c	9.1 c	4.9 a	0.0 a		0.0 a	0.0 a	0.0 b	0.0 a	0.0	0.0 a	0.0 a	0.0 a	0.0 a	0.0 a	0.0 a
Mean	1.9	19.6	10.5	0.4 #		4.9	3.8 *#	3.5	8.9	1.0	6.4 *#	1.3	11.2	9.7	10.6 *	0.8

z Numbers with the same letter/symbol are not significantly different; *P* = 0.05. N indicates the number of mean values included, and – indicates no data. *B. tabaci* abundance (number of insects per plant) and cassava brown streak disease shoot incidences were calculated from annual means throughout the experiment. Plant shoot incidence indicates the percentage of plants with cassava brown streak disease leaf symptoms severity score class of $2. FS, recycled; VT, new.

**Fig. 2:**
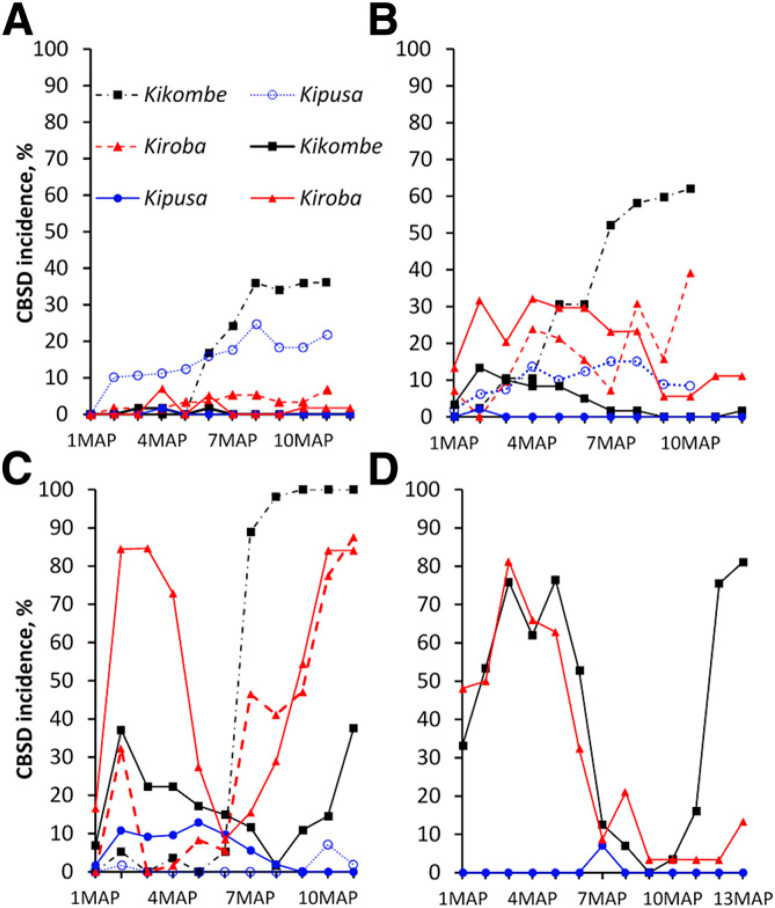
Changes in foliar cassava brown streak disease (CBSD) incidence over time for selected cassava varieties in recycled planting material planted in the Masika (solid lines) and Vuli (dotted lines) seasons from 2013 to 2015 at Chambezi in Bagamoyo, Tanzania. **A**, 2013 to 2014 (Masika) and 2014 to 2015 (Vuli). **B**, 2014 to 2015 (Masika) and 2015 to 2016 (Vuli). **C**, 2015 to 2016 (Masika) and 2016 to 2017 (Vuli). **D**, 2016 to 2017 (Masika). CBSD foliar incidences were calculated as the percentages of plants with symptomatic leaves. CBSD foliar symptoms were observed on Kipusa and cultivars Kikombe and Kiroba, whereas no foliar symptoms were recorded for the others (Chereko, KBH2002_135, Kizimbani, and Mkuranga1). MAP, month after planting.

**Foliar incidence of CBSD**. *Monthly variation of CBSD incidence among cassava varieties during Masika and Vuli growing seasons*. Analysis of changes in plant shoot incidence within season showed a markedly slower rate of increase in Masika than in Vuli. Considering only recycled material, clear differences in the response of the different varieties to CBSD were revealed, and the year × variety × season interaction was significant (*P* < 0.0001). In year 1, Kiroba reached the highest CBSD incidence of 7% in Masika, whereas in the Vuli, Kikombe reached 36% (Fig. 2A). In year 2, Kikombe reached 62% in Vuli, whereas the highest in Masika was Kiroba (32%) (Fig. 2B). By year 3, the incidence of Kikombe reached 100% in Vuli, whereas the highest incidence observed for Kiroba in Masika was 84% (Fig. 2C). The CBSD incidence for Kiroba also increased to 87.6% in Vuli in year 3. In year 4 only, the Masika season was conducted, during which the highest incidence was observed for Kikombe at 81% (Fig. 2D). Overall, plant shoot incidences changed significantly from year 1 (lowest, 0.4%) to year 4 (highest, 10.6%; P < 0.03) in Masika, whereas there were no significant changes in Vuli from year 1 to year 3. Disease progress in Masika seemed to be rapid during the early months of the crop, with the highest incidence at about 3 to 4 MAP, but it dropped sharply to a minimum afterward and then, rose rapidly to a maximum toward the end of the season (Fig. 2C and D). In Vuli, however, disease incidence increased slowly during the early months but progressed rapidly to maxima from 3 to 5 MAP. No significant change in plant shoot incidence was observed during the Vuli season (Fig. 2A to C). In contrast to Kikombe and Kiroba, Kipusa showed little disease progress in Vuli year 1 (an incidence of #25%), and there was no apparent disease progress in the other years. Although there was no difference in plant shoot incidences between recycled and new materials in Vuli, significant differences were observed in Masika (recycled [6.9%] and new [1.5%]; P < 0.0005).

**Fig. 3:**
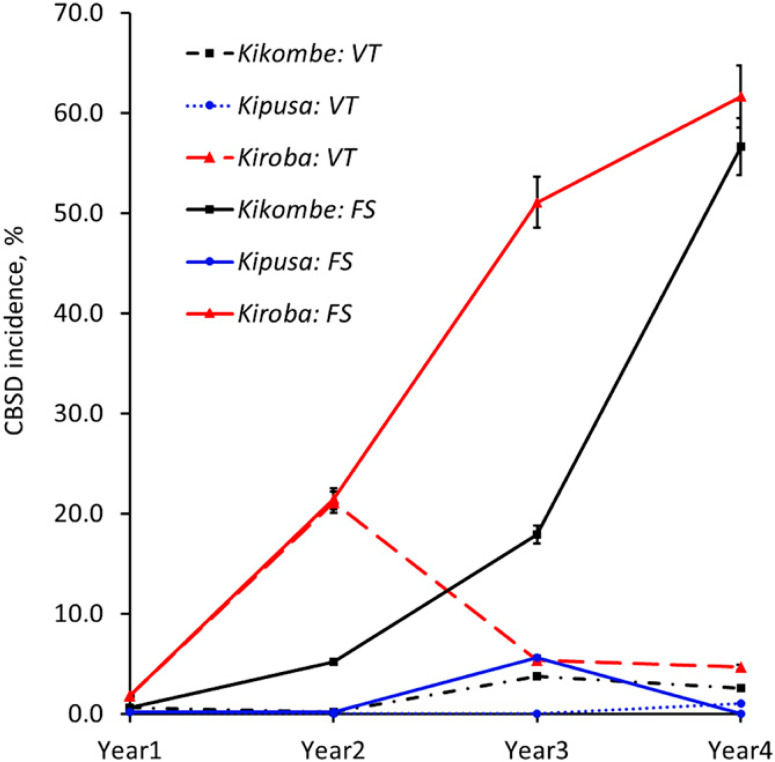
Cassava brown streak disease (CBSD) foliar incidences of selected cassava varieties in recycled (solid lines) and new (dotted lines) planting material planted in the Masika season from 2014 to 2017 at Chambezi in Bagamoyo, Tanzania. CBSD foliar incidences were calculated as the percentages of plants with symptomatic leaves. CBSD foliar symptoms were observed on Kipusa and cultivars Kikombe and Kiroba, whereas no foliar symptoms were recorded for the others (Chereko, KBH2002_135, Kizimbani, and Mkuranga1). Error bars represent percentage errors. FS, recycled; VT, new.

*CBSD foliar incidence on new versus recycled planting materials*. Results showed significant (*P* < 0.0001) interaction effects between rainy season and variety for CBSD shoot symptoms, for which year effect was also significant (*P* = 0.03). For the recycled material, plant shoot incidence was low (5.2%) in year 2, but it increased significantly toward year 4 (42.3%, *P* < 0.0001) for Kikombe in Masika (Table 1). However, significant increases (*P* < 0.04) in plant shoot incidence for years 1 (3.2%), 2 (17.4%), and 3 (31.6%) were observed for recycled Kiroba in the Vuli season (Table 1). For Kipusa, plant shoot incidence rose to ~5% by year 4 in the Masika season, whereas it rose to 14% in year 1 in Vuli and dropped to 1.0% in Vuli year 3 (Table 1), although these changes were statistically insignificant. Significantly lower (*P* < 0.002) plant shoot incidence was observed in new material overall (mean = 3.3%) compared with recycled materials in Masika (mean = 8.2%) (Fig. 3), although the variety × planting material interaction was not significant. Whereas overall plant shoot incidences for both recycled and new material were similar in the Vuli season for Kikombe and Kipusa, there were significant differences (*P* < 0.04) for the tolerant variety Kiroba: 24.5% (recycled) and 3.2% (new). In the new material, Kiroba had the highest plant shoot incidence (21.2%) (Table 1) in Masika year 2, but it declined gradually in years 3 and 4, whereas for Kikombe, the highest incidence in new material in Masika was recorded in year 4 (Fig. 3 and Table 1). Kipusa remained free of symptoms throughout the 3 years in the new category, but there was a small rise in incidence (<1%) for year 4 in Masika. Apparently, the interaction variety × rainy season × planting material selection had no effect on plant shoot incidence.

*CBSD incidence among different generations of recycled cassava planting material.* Overall, plant shoot incidences were significantly lower in Masika (3.8%) than in Vuli (8.9%, P < 0.008) (Table 1). Significant differences were also observed among the varieties tested, with Kikombe, Kiroba, and Kipusa expressing shoot symptoms. Varieties Chereko, KBH2002_135, Kizimbani, and Mkuranga1 did not express CBSD symptoms in all the seasons, except in Masika year 4 (Table 1) (*P* < 0.008). Overall, plant shoot incidence significantly increased over the course of the four Masika seasons, with the lowest incidence in year 1 (0.9%) and the highest incidence in year 4 (24.6%, *P* < 0.0003), although the changes from year 1 to year 2 (from 0.9 to 9.0%) and from year 3 to year 4 (from 14.9 to 24.6%) were not significantly different. In contrast, the overall change in plant shoot incidences in the Vuli season was not significant over the course of the 3 years. Of the varieties that had leaf symptoms, Kipusa (1.7%) had the lowest overall mean CBSD shoot incidence in Masika (P < 0.0005) compared with Kikombe (16.5%) and Kiroba (18.8%). In Vuli, Kikombe (32.2%) had the highest mean plant shoot incidence (P < 0.005) compared with Kipusa (17.4%) and Kiroba (8.6%) (Table 2).

**Table 2:** Cassava brown streak disease (CBSD) shoot and root incidences of recycled planting material for Kipusa and cultivars Kiroba and Kikombe planted during the Masika season at Chambezi, Bagamoyo, Tanzania from 2014 to 2017^z^

Variety and year	*N*	Plant total incidence	Plant shoot incidence	Plant root incidence	Root incidence	Cross-sectional root cuts incidence	Unusable roots incidence	Root severity
Kiroba								
1	3	3.3 c	1.8	2.1	0.3	0.1	0.0	2.00 a
2	3	48.3 b	21.5	15.8	3.8	1.9	0.2	2.05 a
3	3	88.0 a	21.1	12.2	2.4	0.9	1.4	2.72 a
4	3	80.5 a	30.8	11.8	5.3	2.9	3.5	2.88 a
Mean/total	12	55.0	18.8	10.5	3.0	1.5	1.3	2.41
Kipusa								
1	3	36.4	0.2	36.4	7.4	6.2	3.2	2.25 a
2	3	11.3	0.2	9.0	2.1	1.2	1.1	2.20 a
3	3	30.8	5.6	26.4	7.8	6.4	3.3	2.13 a
4	3	21.9	0.6	21.9	9.4	4.9	3.7	2.12 a
Mean/total	12	25.1	1.7	23.4	6.7	4.7	2.8	2.18
Kikombe								
1	3	5.2 b	0.6 c	3.6 b	0.8 b	0.5 c	0.5 c	3.50 a
2	3	25.0 b	5.2 c	12.1 b	3.4 b	3.3 c	2.6 c	2.61 a
3	3	63.1 a	17.9 b	53.1 a	26.0 a	14.3 b	18.9 b	3.18 a
4	3	94.0 a	42.2 a	61.3 a	40.0 a	24.5 a	36.1 a	3.90 a
Mean/total	12	46.8	16.5	32.5	17.6	10.7	14.5	3.3

z Values with the same letter are not significantly different; *P* = 0.05. *N* indicates the number of replication plots. Plant total incidence is the percentage of plants with CBSD symptoms in either shoots or roots. Plant shoot incidence is the percentage of plants with CBSD leaf symptoms severity score class of $2. Plant root incidence is the percentage of plants with one or more roots with severity score class of $2 in one or more of the five cross-sectional cuts made through the root. Root incidence is the percentage of roots with CBSD severity score class of $2. Cross-sectional cuts root incidence is the percentage of cross-sectional cuts with severity score class of $2. Unusable root incidence is the percentage of roots with at least one cut with severity score of $3. Root severity is the average severity of CBSD symptoms in cross-sectional cuts showing symptoms (i.e., cuts with severity scores 2 to 5).

**Fig. 4:**
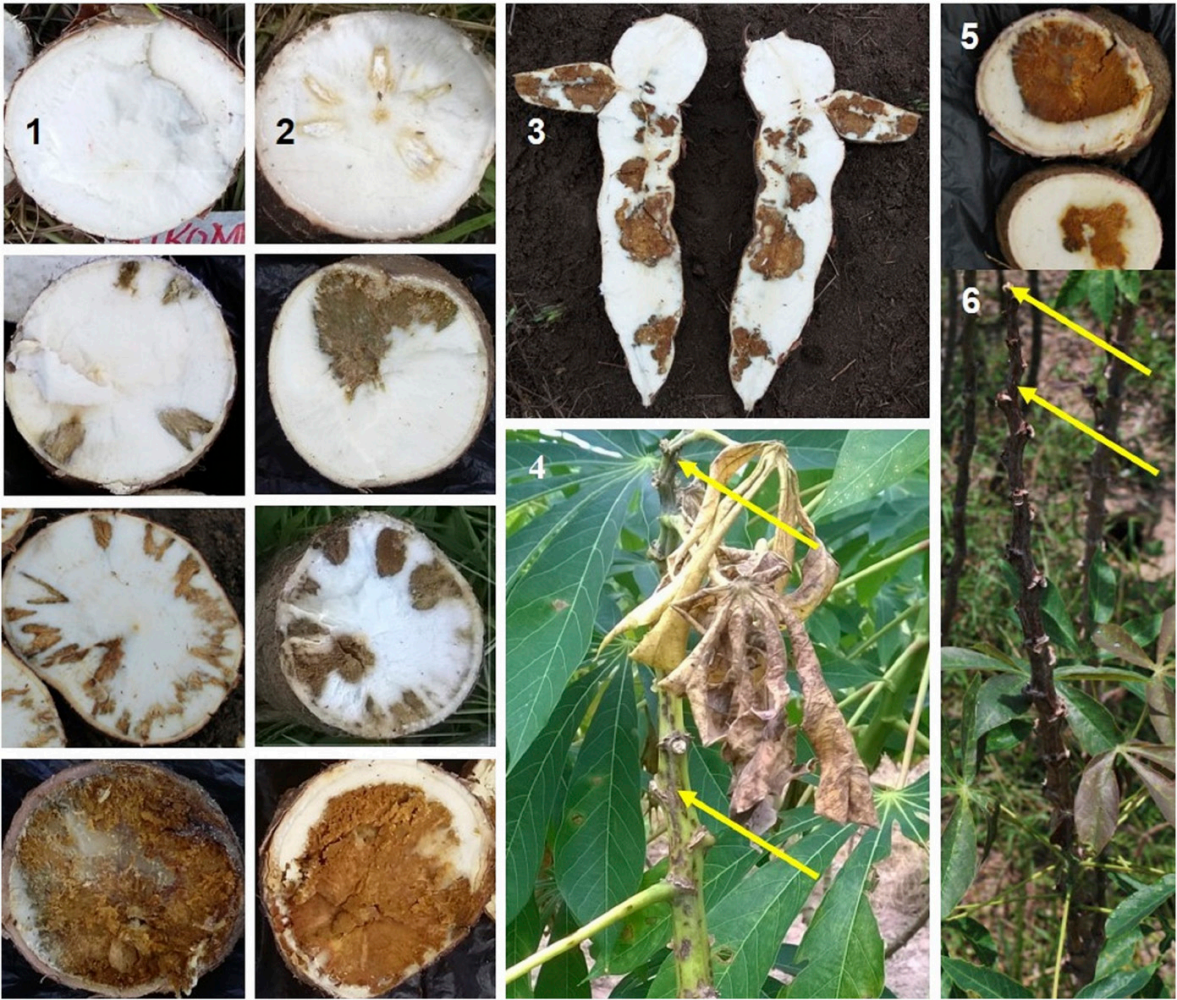
Cassava brown streak disease (CBSD) symptoms on cassava variety Kipusa and cultivars Kikombe and Kiroba. Stem necrotic lesions and shoot dieback are indicated by arrows. **1**, CBSD root necrotic symptoms observed through cross-sectional cuts on roots of Kikombe. **2**, CBSD root necrotic symptoms observed through cross-sectional cuts on roots of Kipusa. **3**, CBSD root necrotic symptoms observed through a longitudinal cut on roots of Kikombe. **4**, CBSD foliar symptoms expressed in Kikombe. **5**, CBSD root necrotic symptoms observed through cross-sectional cuts on roots of Kiroba. **6**, CBSD foliar symptoms expressed in Kiroba.

**Yield and CBSD root symptoms assessment: CBSD root symptoms**. Root necrosis symptoms were observed in all cassava varieties evaluated. Although there were several roots with severity scores class 3 (moderate severity), the symptoms observed for most of the varieties were mild (severity score was <3). In contrast, severe to very severe symptoms (score$3) were observed for Kikombe and Kipusa (Fig. 4). Interestingly, Kiroba, which is considered to be tolerant to CBSD, had severe root necrosis symptoms (up to severity score class of 5) (Fig. 4) in Masika year 4, albeit with low incidence (5.3%) (Table 2). Kikombe had a significant increase from Masika year 2 to year 3 in plant root incidence (*P* < 0.01) and root incidence (*P* < 0.001) (Table 2), whereas there were significant increases from year 2 to year 4 in cross-sectional root cuts incidence (*P* < 0.001) and unusable roots incidence (P < 0.001). Kikombe, however, had the highest overall CBSD root severity (3.26, *P* < 0.0001) (Table 3). In general, Kikombe had the highest incidences overall for all root incidence types, although it had plant total incidence similar to Kiroba (*P* < 0.0001) (Table 3). An overall assessment of all treatments showed significant differences in plant total incidence (*P* < 0.012) between recycled (26.6%) and new materials (17.2%). Differences between these treatments were, however, not significant for plant root incidence, root incidence, cross-sectional root cuts incidence, or unusable roots incidence. Furthermore, no significant differences were observed between theMasika and Vuli seasons in terms of overall plant total and plant root incidences. However, significant differences were observed between Masika and Vuli seasons for root incidence (4.4 and 10.3%, respectively; *P* < 0.005), cross-sectional root cuts incidence (2.6 and 9.6%, respectively; *P* < 0.0001), and unusable roots incidence (2.4 and 8.5%, respectively; *P* < 0.001). Linear regression analysis showed significant associations between CBSD root severity and plant total incidence (*t* = 2.5, P = 0.013), plant root incidence (*t* = 5.28, *P* < 0.0001), root incidence (*t* = −4.89, *P* < 0.0001), and unusable root incidence (*t* = 4.79, *P* < 0.0001). By contrast, for CBSD shoot severity, a significant regression relationship was only demonstrated with plant total incidence (*t* = 2.05, *P* < 0.041) and cross-sectional root cuts incidence (t = 2.62, P < 0.01) in the recycled material.

**Table 3:** Cassava brown streak disease (CBSD) root severity and incidences of selected varieties planted during the Masika and Vuli seasons at Chambezi, Bagamoyo, Tanzania from 2013 to 2017^z^

Variety	*N*	Plant total incidence	Plant root incidence	Root incidence	Cross-sectional root cuts incidence	Unusable roots incidence	Root severity
Chereko	33	9.3 bc	9.3 c	2.7 b	1.4 b	0.6 b	2.09 b
KBH2002_135	33	7.5 bc	7.5 c	2.0 b	1.4 b	0.8 b	2.30 b
Kikombe	33	53.8 a	44.7 a	30.7 a	24.4 a	26.1 a	3.26 a
Kipusa	35	21.2 b	19.4 b	7.6 b	6.4 b	5.0 b	2.47 b
Cultivar Kiroba	36	43.7 a	6.8 c	1.8 b	1.8 b	0.7 b	2.47 b
Kizimbani	33	7.3 bc	7.3 c	1.3 b	0.8 b	0.2 b	2.49 b
Mkuranga1	36	5.7 c	5.9 c	1.5 b	1.5 b	0.6 b	2.30 b
Mean/total	239	21.2	14.4	6.8	5.4	4.8	2.48

z Values with the same symbol are not significantly different. *N* indicates the number of mean values included; *P* = 0.05. Plant total incidence is the percentage of plants with CBSD symptoms in either shoots or roots. Plant root incidence is the percentage of plants with one or more roots with severity score class of $2in one or more of the five cross-sectional cuts made through the root. Root incidence is the percentage of roots with CBSD severity score class of $2. Cross-sectional root cuts incidence is the percentage of cross-sectional cuts with severity score class of $2. Unusable root incidence is the percentage of roots with at least one cut with severity score of $3. Root severity is the average severity of CBSD symptoms in cross-sectional cuts showing symptoms (i.e., cuts with severity scores 2 to 5).

CBSD root incidences were significantly higher overall (all treatments and varieties put together) in Vuli than in Masika: root incidence (11.5 and 5.3%, respectively; *P* < 0.033), cross-sectional root cuts incidence (9.9 and 3.1%, respectively; *P* < 0.005), and unusable roots incidence (9.3 and 3.1%, respectively; *P* < 0.016). For plant root incidence, Kikombe (44.7%) had the highest (*P* < 0.0001) followed by Kipusa (19.4%) and Chereko (9.3%). The remaining varieties were similar to Chereko, with plant root incidences ranging from 7.5% (KBH2002/135) to 5.9% (Mkuranga1) (Table 3). All varieties had similar cross-sectional root cuts and unusable roots incidences, but Kikombe had the highest (*P* < 0.0001) (Table 3). In the recycled material, Kikombe (59.2%) and Kiroba (50.5%) had similar plant total incidences followed by Kipusa (26.8%) and KBH2002_135 (10.0%), which was similar to the other varieties (*P* < 0.0001). Kikombe (49.6%) also had the highest plant root incidence followed by Kipusa (24.5%) and KBH2002_135 (10.0%), which was similar to the other varieties (P < 0.0001). Furthermore, Kikombe had the highest root incidence (35.1%), crosssectional root cuts incidence (27.3%), and unusable roots incidence (30.1%) overall in the recycled material, whereas the remaining varieties were similar (P < 0.0001), and Kizimbani (0.3%) had the lowest. These values were similar to the overall values observed (Table 3). No significant differences were observed for Kipusa for any of the root incidence types. For Kiroba, no significant changes in CBSD root incidences were observed throughout the study period during the Masika season, but in Vuli, plant total incidence increased significantly (P < 0.002) from year 2 (32.1%) to year 3 (87.6%), although year 1 (13.9%) was similar to year 2 (Table 4). In the Vuli season, there were significant increases from year 1 to year 3 for Kikombe (plant root incidence [*P* < 0.055] and root incidence [*P* < 0.044]), whereas unusable root incidence increased significantly from year 1 to year 2 (Table 4). AUDPC showed significant differences among varieties compared. Kikombe (57.9% pcu) had the highest AUDPC (*P* = 0.04) followed by Kipusa (21.5% pcu) and Kiroba (2.1% pcu). Vuli season (42.3% pcu) had significantly (*P* = 0.04) higher overall AUDPC thanMasika season (12.0% pcu) for these three varieties. Both the interactions variety × season (P = 0.005) and variety × planting material (*P* = 0.04) were significant for AUDPC. These results suggest that the rate of increase in unusable CBSD root incidence is mostly affected by the factors variety and planting season.

**Table 4:** Cassava brown streak disease (CBSD) shoot and root incidences of recycled planting material for Kipusa and cultivars Kiroba and Kikombe planted during the Vuli season at Chambezi, Bagamoyo, Tanzania from 2014 to 2017^y^

Variety and year	*N*	Plant total incidence	Plant shoot incidence	Plant root incidence	Root incidence	Cross-sectional root cuts incidence	Unusable roots incidence	Root severity
Kiroba								
1	3	13.9 b	3.2 b	10.3 a	3.7 a	2.8 a	1.9 a	2.13 a
2	3	32.1 b	17.4 ab	0.0 a	0.0 a	0.0 a	0.0 a	-^z^
3	3	87.6 a	31.6 a	1.7 a	0.6 a	1.0 a	0.3 a	2.33 a
Mean/total	9	44.5	17.4	4.0	1.4	1.3	0.7	2.2
Kipusa								
1	3	51.0 a	14.7 a	45.8 a	32.8 a	31.3 a	29.8 a	3.96 a
2	3	32.6 a	10.2 a	26.2 a	8.9 a	8.9 a	6.4 a	3.28 a
3	3	3.6 a	1.0 a	5.9 a	0.9 a	0.4 a	0.0 a	2.00 a
Mean/total	9	29.1	8.6	26.0	14.2	13.5	12.1	3.1
Kikombe								
1	3	43.2 a	16.6 a	39.9 b	24.5 b	20.9 a	17.0 b	2.80 a
2	3	83.9 a	34.5 a	77.3 ab	68.7 ab	59.2 a	65.2 a	3.79 a
3	3	100.0 a	45.6 a	100.0 a	82.0 a	68.5 a	70.5 a	3.30 a
Mean/total	9	75.7	32.2	72.4	58.4	49.5	50.9	3.3

y Values with the same symbol are not significantly different; *P* = 0.05. *N* indicates the number of replication plots. Plant total incidence is the percentage of plants with CBSD symptoms in either shoots or roots. Plant shoot incidence is the percentage of plants with CBSD leaf symptoms severity score class of $2. Plant root incidence is the percentage of plants with one or more roots with severity score class of $2 in one or more of the five cross-sectional cuts made through the root. Root incidence is the percentage of roots with CBSD severity score class of $2. Cross-sectional cuts root incidence is the percentage of cross-sectional cuts with severity score class of $2. Unusable root incidence is the percentage of roots with at least one cut with severity score of $3. Root severity is the average severity of CBSD symptoms in cross-sectional cuts showing symptoms (i.e., cuts with severity scores 2 to 5).

z -No root necrosis symptoms observed.

**Shoot and tuberous root yield, DM content, and HI.** Overall mean root yield ranged from 31.4 t/ha (Kizimbani) to 21.3 t/ha (Kikombe and KBH2002_135; *P* < 0.0001), with overall mean root yield being significantly higher in Masika (29.4 t/ha) than in Vuli (21.2 t/ha, *P* < 0.0001).

*Effect of CBSD on root yield of cassava varieties during Masika and Vuli.* Significantly higher harvests of marketable root yield were obtained in Masika (28.9 t/ha) than in Vuli (20.9 t/ha, *P* < 0.0001). There were significant annual root yield differences (*P* < 0.0002) over the course of the experiment in Masika, where year 4 had the lowest (23.8 t/ha), whereas year 2 had the highest (36.9 t/ha, *P* < 0.0001). For the Vuli experiments, year 3 had the lowest (16.4 t /ha), which was significantly different from year 1 (23.4 t/ha) and year 2 (27.5 t/ha, *P* < 0.0002). Root yield differences between new and recycled materials were not significant in either season (Table 5). Kikombe had the highest percentage (26.5%) of unmarketable roots overall followed by Kipusa (5.0%), whereas the remaining varieties had <1.0% (*P* < 0.0001). Furthermore, Kikombe had the lowest marketable yields of all varieties in Masika (21.2 t/ha, *P* < 0.0004) and Vuli (8.6 t/ha, *P* < 0.0007), whereas Kizimbani had the highest: 35.5 t/ha (Masika), and Kipusa had 29.6 t/ha (Vuli).

**Table 5:** Overall yield changes over time in cassava brown streak disease-affected cassava Kipusa and cultivars Kiroba and Kikombe planted during the Masika and Vuli seasons at Chambezi, Bagamoyo, Tanzania from 2013 to 2017^z^

Year	Root yield (t/ha)				Marketable yield (t/ha)			
	Masika		Vuli		Masika		Vuli	
	FS	VT	FS	VT	FS	VT	FS	VT
1	31.5 ab	31.5 a	30.1 a	24.3 a	31.1 ab	31.1 a	20.8 a	20.8 a
2	34.6 a	31.3 a	24.3 a	36.7 a	34.3 a	31.2 a	22.9 a	36.0 a
3	24.4 ab	31.9 a	16.9 b	17.9 a	22.7 bc	31.5 a	14.3 a	15.0 a
4	21.6 b	25.3 a	–	–	19.4 c	24.1 a	–	–

z The figures presented in the table are means across years; – indicates that the experiment was not conducted for Vuli in year 4. Values with the same letter are not significantly different; *P* = 0.05. FS, recycled; VT, new.

**Fig. 5:**
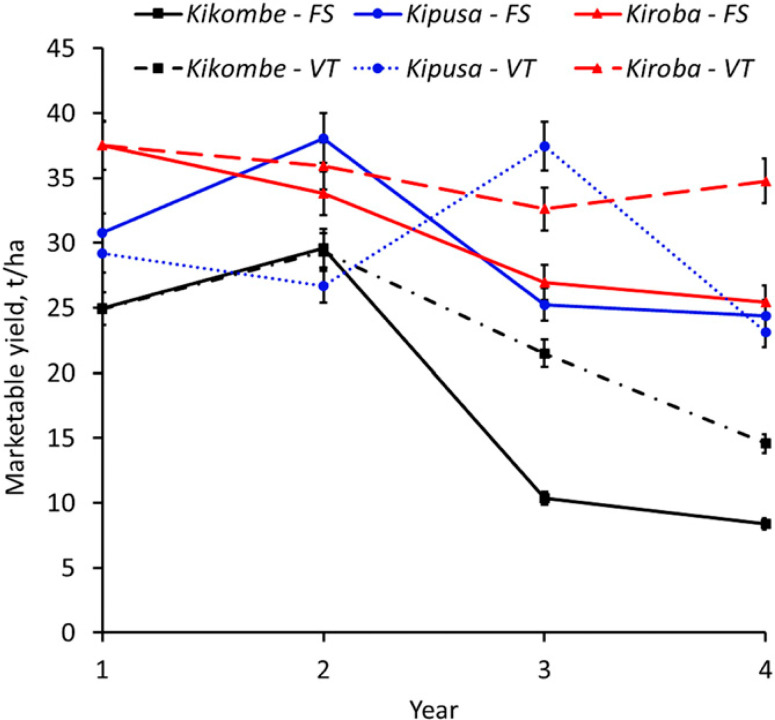
Change in marketable yield over time of selected cassava varieties in recycled (solid lines) and new (dotted lines) planting material planted in the Masika season from 2013 to 2017 at Chambezi in Bagamoyo, Tanzania. Marketable yield (tons per hectare) was the total fresh root yield minus unusable root yield (roots with root necrosis severity scores of $3). Significant reductions in marketable yield over time were observed only in those that had foliar symptoms (Kipusa and cultivars Kiroba and Kikombe). Error bars represent standard errors. FS, recycled; VT, new.

**Fig. 6:**
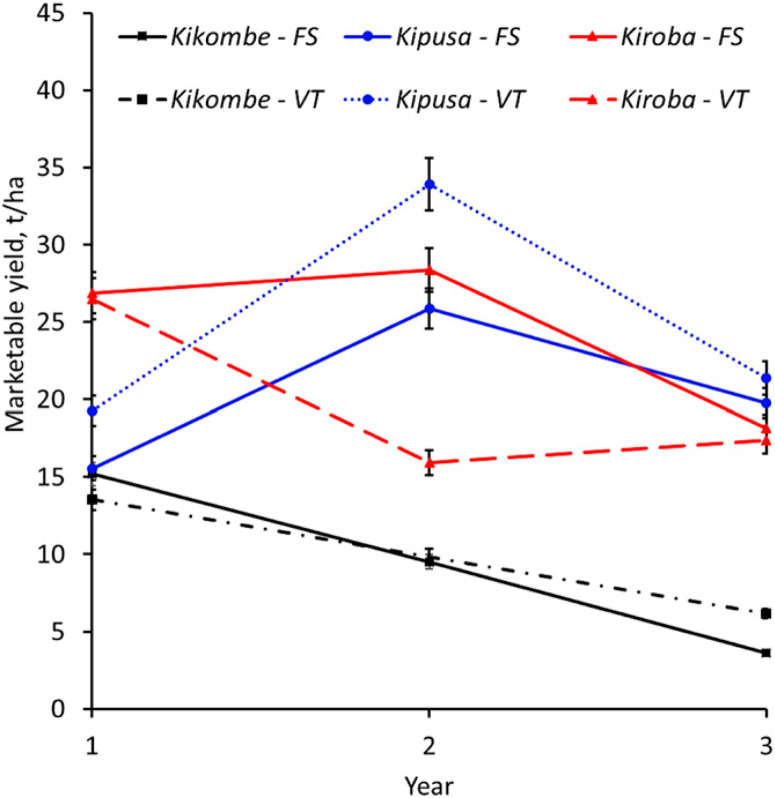
Change in marketable yield over time of selected cassava varieties in recycled (solid lines) and new (dotted lines) planting material planted in the Vuli season from 2014 to 2017 at Chambezi in Bagamoyo, Tanzania. Marketable yield (tons per hectare) was the total fresh root yield minus unusable root yield (roots with root necrosis severity scores of $3). Significant reductions in marketable yield over time were observed only in those that had foliar symptoms (Kipusa and cultivars Kiroba and Kikombe). Error bars represent standard errors. FS, recycled; VT, new.

Comparison of yield over time between the two seasons showed that, at the end of year 3, there were significant differences in root yield (means: 27.3 t/ha in Masika, 18.2 t/ha in Vuli; *P* < 0.011) for Kiroba. For Kikombe, however, significant differences were only observed for shoot yield (means: 26.7 t/ha in Masika, 8.3 t/ha in Vuli). No significant differences were observed for the other varieties. However, at the outset of the experiments, differences in root yield between Masika and Vuli were not significant for any of the tested varieties. Significant reductions in marketable yield over time were observed in both Masika and Vuli, with the lowest overall means in Masika year 4 (19.4 t/ha) and Vuli year 3 (14.3 t/ha) for recycled materials, whereas marketable yield in new materials was similar throughout the experiment (Table 5). Varieties also differed significantly in the rate of reduction in marketable yield over time. Although the least affected varieties showed no significant decrease in marketable yield over time, the most affected Kikombe had the greatest decrease in marketable yield, whereas in Masika, marketable yield decreased from 25.1 t/ha at the start of the experiment to 8.3 t/ha in year 4 (*P* = 0.05) (Fig. 5). Similarly, in Vuli, marketable yield for Kikombe decreased from 15.2 t/ha in year 1 to 3.6 t/ha in year 3 (*P* = 0.05) (Fig. 6). Analysis of AUYPC showed that variety (*P* = 0.02) and rainy season (*P* = 0.002) effects and the interaction variety × rainy season (*P* = 0.0004) were significant for AUYPC, whereas the interaction year × variety × rainy season × planting material was not.

*Effect of CBSD on shoot yield of cassava varieties*. Overall, mean shoot yield was higher in new (27.1 t/ha) than recycled materials (25.2 t/ha, *P* < 0.04). However, no significant differences were observed between the Masika and Vuli seasons. The variety KBH2002_135 had the highest shoot yield (30.0 t/ha), whereas Chereko had the lowest (23.1 t/ha, P < 0.0001). There was a large reduction in shoot yield from year 2 (37.8 t/ha) to year 4 (13.4 t/ha, *P* < 0.0001) in Masika, although year 1 (37.4 t/ha) was similar to year 2. Similarly, in Vuli, year 1 (39.6 t/ha) was similar to year 2 (40.7 t/ha), but a significant reduction in shoot yield was seen from year 2 to year 3 (13.7 t/ha, *P* < 0.0001).

Yield reductions over time between the two seasons showed that Masika had significantly higher shoot yields than Vuli at the end of year 3 (means: 13.2 t/ha in Masika and 7.9 t/ha in Vuli; P < 0.023 for Kiroba and means: 26.7 t/ha in Masika and 8.3 t/ha Vuli for Kikombe). No significant differences were observed for the other varieties. However, at the outset of the experiments, differences in shoot yield between Masika and Vuli were not significant for any of the tested varieties.

*Effect of CBSD on DM content and HI of different cassava varieties*. Significant differences were observed in DM among all tested varieties, where Kipusa (35.0%) had the highest overall and Kiroba had the lowest (32.7%, *P* < 0.001). Moreover, higher DM was recorded for new (34.1%) than recycled material (33.2%) overall (P < 0.002).Variety (*P* = 0.0006) and year (*P* = 0.04) effects were significant for DM. The interactions year × variety (P < 0.0001), year × season (P = 0.02), and variety × season (*P* = 0.0004) were significant for DM. Root HI was significantly higher in Masika (54.5%) compared with Vuli (47.5%, *P* < 0.044), whereas significant differences were also observed among varieties (*P* < 0.001). The highest overall HI (57.3%) was recorded for Kiroba, whereas the lowest (42.9%) was recorded for KBH2002_135. In addition to the interaction effects of year × variety (*P* < 0.05), year × season (*P* = 0.0002), and variety × season (*P* < 0.0001), unlike for DM, the interactions variety × planting material (*P* = 0.05), season × planting material (*P* = 0.008), and year × season × planting material (*P* = 0.02) were significant for HI.

*Relationship among CBSD shoot and root incidence and severity versus root and shoot yield*. Weak negative associations were realized between plant shoot incidence, root yield (*r* = −0.23, P < 0.0001), and shoot yield (*r* = −0.16, *P* = 0.013) (Table 6). A strongly positive correlation between root incidence and unusable roots incidence was observed overall (r = 0.98, P < 0.0001). There was, however, no association between root incidence and shoot yield. Shoot yield was associated with root yield (*r* = 0.46, *P* < 0.0001), but a strongly negative correlation was observed between root incidence and marketable yield (*r* = −0.98, *P* < 0.0001). CBSD root severity was negatively correlated with root yield (*r* = −0.40, *P* < 0.0001) but weakly correlated to shoot yield (*r* = −0.22, *P* = 0.01). CBSD root severity had a strong negative association with marketable yield (*r* = −0.54, *P* < 0.0001) but a moderately positive correlation to root incidence (r = 0.48, P < 0.0001). Furthermore, CBSD plant shoot incidence was negatively correlated to marketable root yield (r = −0.73, P < 0.0001).

**Table 6:** Pearson correlation analysis of the effects of cassava brown streak disease (CBSD) shoot incidence, CBSD root severity, CBSD shoot severity, and root incidence on cassava root and shoot yieldsz

Effective parameter	Root yield	Shoot yield	Marketable yield	Unusable root incidence	Root incidence
Shoot CBSD incidence	−0.23 (*P* = 0.001)	−0.16 (*P* = 0.013)	−0.73 (*P* < 0.0001)	0.73 (*P* < 0.000)	0.71 (*P* < 0.0001)
Root CBSD severity	−0.40 (*P* = 0.0001)	−0.22 (*P* = 0.01)	−0.54 (*P* < 0.0001)	0.54 (*P* < 0.0001)	0.48 (*P* < 0.0001)
Shoot CBSD severity	−0.16 (*P* = 0.012)	−0.12 (*P* = 0.06)	−0.56 (*P* < 0.0001)	0.58 (*P* < 0.0001)	0.58 (*P* < 0.0001)
Root incidence	−0.20 (*P* = 0.002)	−0.09 (*P* = 0.16)	−0.98 (*P* < 0.0001)	0.98 (*P* < 0.0001)	
Shoot yield	0.46 (P < 0.0001)		0.11 (*P* = 0.11)	−0.11 (*P* = 0.11)	−0.09 (*P* = 0.16)

^z^ Pearson correlation coefficients, *N* = 231. Probability > |*r*| under H0: Rho = 0.

**Surrounding disease and vector abundance.** The level of disease inoculum surrounding the experimental plot was assessed using three elements: (i) surrounding CBSD pressure measured as the sum of the CBSD pressure of all fields surrounding the experimental plot (Legg et al. 1997), (ii) total surrounding CBSD incidence and severity, and (iii) vector abundance in the fields surrounding the experimental plot. Surrounding CBSD pressure increased from year 2 (15.7) to year 3 (46.6) in the Masika season. Vuli year 1 had the highest CBSD pressure (78.9), but this declined to 36.1 in year 2. *B. tabaci* abundance was low for all years in surrounding fields in the Masika and Vuli seasons. The highest abundance was recorded surrounding the experiment in Masika year 2 (1.3 insects per plant), although this is regarded as low (Legg et al. 2011). The low surrounding vector abundance observed was presumably owing to the relatively older plants (6 to 10 MAP; data not shown) encountered during inoculum pressure assessment. This crop age is not the most suitable growth stage for high whitefly numbers. Average surrounding CBSD severities in Masika (3.3) and Vuli (3.0) were similar. Overall surrounding CBSD incidence in Masika was 36.8%, whereas in Vuli, it was 24.5%. Correlation analyses revealed no significant associations between the surrounding CBSD pressure and disease progress within the experimental plots over the entire duration of this study. These results indicate that, whereas initial spread depends on surrounding inoculum, differences in disease progress within the experimental plots during Masika are rather as a result of within-field transmission.

**Fig. 7:**
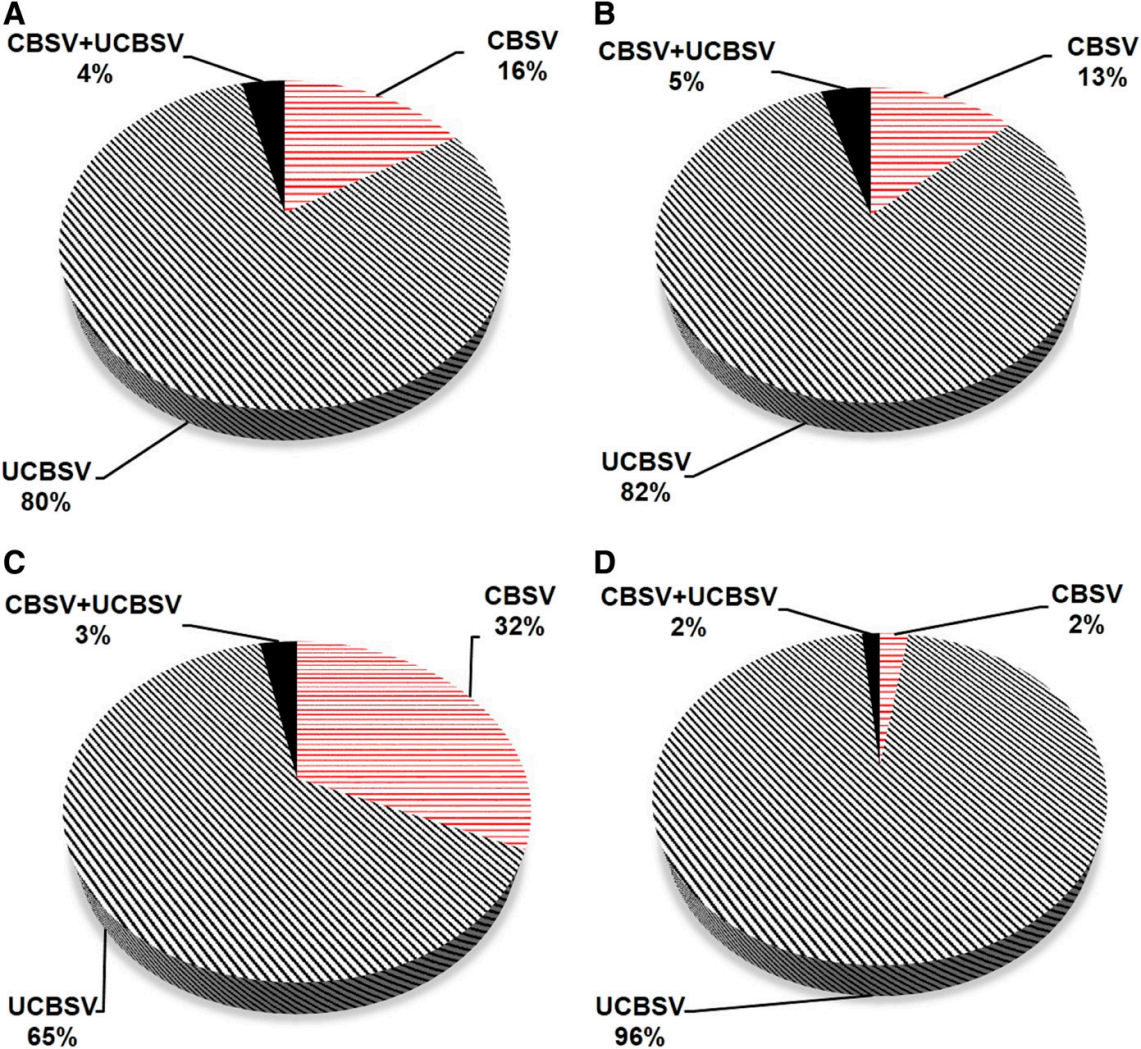
Proportion of cassava brown streak ipomovirus (CBSI) species detected in the Masika and Vuli seasons at Chambezi, Bagamoyo, Tanzania from 2014 to 2017. Virus testing was conducted using *Cassava brown streak virus* (CBSV)-and *Ugandan cassava brown streak virus* (UCBSV)-specific TaqMan assays (Adams et al. 2013; Shirima et al. 2017). Leaf samples collected from 2014 to 2017 for Masika (years 2 to 4) and Vuli (years 1 to 3) were tested. **A**, Proportion of detected CBSIs in recycled material in Masika. **B**, Proportion of detected CBSIs in recycled material in Vuli. **C**, Proportion of detected CBSIs in new material in Masika. **D**, Proportion of detected CBSIs in new material in Vuli.

**Real-time RT-PCR detection of CBSIs.** Approximately 1,000 cassava leaf samples (about 140 samples for each of the seven varieties) were tested per year for each of the Masika and Vuli seasons planted. Collectively, using only the group of recycled material, UCBSV had the largest proportion (80%) of the positive tests throughout the Masika season. CBSV was present in 16% of positive samples, whereas mixed infections (CBSV and UCBSV) were rare (4%) in Masika. Similarly, in Vuli, UCBSV was most frequent (82%) followed by CBSV (13%) and mixed infections (5%) (Fig. 7). Moreover, the proportions of the viruses detected in the new treatment plots in Masika and Vuli were similar. Considering all tested samples, the overall test results showed that UCBSV had 15.9%, CBSV had 3.2%, and mixed infections had 0.7% of tested plants in Masika, whereas in Vuli, UCBSV had 12.3%, CBSV had 2.0%, and mixed infections had 0.7%. In Masika, the largest difference was observed for UCBSV, where Kikombe (51.4%) and Kiroba (51.4%) had the highest proportions of all detected CBSIs, whereas the other varieties had between 3.7% (Kipusa) and 0.2% (Kizimbani; *P* < 0.0001). No significant differences were observed for CBSV and mixed infections among varieties in Masika. Only the CBSV percentage of positive samples changed significantly over the course of the Masika experiments, where year 3 had the highest value (8.7%). In Vuli, significant differences were also observed for UCBSV, where Kikombe (41.2%) had the highest proportion of positive samples followed by Kiroba (29.9%), whereas the other varieties had between 4.7% (KBH2002_135) and 1.4% (Mkuranga1; *P* < 0.0001). Kipusa (10.8%) had the highest proportion of samples testing positive for CBSV in Vuli followed by Kiroba (4.0%) and the other varieties (*P* < 0.008). No differences were significant among varieties for mixed infections. None of the differences in the proportion of infection by the two CBSI species were significant comparing the Masika and Vuli seasons. However, significant overall differences were observed in the proportions of detected CBSIs between the different varieties. CBSV was the highest in Kipusa (6.8%, *P* < 0.006) followed by Kiroba (3.2%) and the other varieties (<2.0%). UCBSV was the highest (*P* < 0.0001) in Kikombe (36.9%) and Kiroba (35.7%) followed by the other varieties (<3.0%). The highest proportion (2.4%, *P* = 0.001) of mixed infections was observed for Kiroba. Furthermore, changes in the proportions of detected CBSIs over the course of the Vuli season were insignificant.

## Discussion

CBSD is the most devastating of the biotic stresses affecting cassava production in East and Central Africa. Over the past decade, most attention has been focused on host resistance for the management of cassava virus diseases. However, the effects of CBSD continue to adversely affect the livelihoods of many farmers, because no varieties with durable resistance have been identified. Five elite cassava varieties obtained from each of five countries in East and Southern Africa were pooled for virus “clean up,” and planting materials for the total set of 25 were then multiplied and sent back to each of the five countries for multilocational evaluation (IITA 2012). Although several varieties were shown to be moderately resistant in each of the countries (Tumwegamire et al. 2018), all became affected to some degree by CBSD—a situation that highlights the need to focus additionally on field management measures. A novel approach using community phytosanitation for the management of CBSD in the Lake Victoria and coastal zones of Tanzania (Legg et al. 2017) demonstrated disease management and yield benefits for farmers as a result of working together to remove infected crops and replacing these with near virus-free planting material of improved varieties. Although longer-term management programs are being implemented, including the search for stronger and more durable CBSD resistance, this study focused on evaluating the effects of recycling planting material in cassava cropping systems. This was justified by the fact that farmers reuse their planting material over several planting cycles, because new quality seed is not readily available to them and may prove uneconomic (Gildemacher et al. 2011). Additionally, there was a requirement to understand how durable some of the most resistant varieties would be over repeated cycles of planting and replanting. The study site was located in an area with a bimodal rainfall regimen (coastal Tanzania); therefore, two experiments were run concurrently. A farmer participated in the selection of planting material for the recycled plantings to mimic a real “farmer-situation.” Results from this study revealed considerable differences between growing seasons and the response of different varieties to the effects of disease over repeated planting cycles.

Vector abundance was greater in the Vuli season, resulting in generally higher CBSD incidence. The high contrast in the rates and patterns of disease spread between the two seasons used in this study resulted primarily from the higher abundance of *B. tabaci* in the Vuli season (mean > 19 insects per plant) than in the Masika season (mean < 2 insects per plant). More than three cassava *B. tabaci* adults per plant has previously been considered to be “high” (Legg et al. 2011) and sufficient to cause significant spread of CBSIs. Plants growing in Masika were colonized by low *B. tabaci* numbers throughout the growth cycle. Although the older crop growth stages went through the warmer and humid months of December to March when *B. tabaci* numbers are typically higher, whitefly numbers remained low, almost certainly because these insects do not prefer older cassava plants (Fishpool et al. 1995; Legg 1995). In contrast, plants planted in the Vuli season had the young crop stage growing through a period of heavy vector infestation during the months from December to March, making these plants more vulnerable to the effects of disease owing to heavy vector infestation (Moriones et al. 1998). The combination of high vector abundance occurring during the vulnerable early growth stage of cassava plants during the Vuli season explains the significantly greater incidence of CBSD recorded in that season compared with Masika. By contrast, the relative absence of vectors during the early growth stages of the Masika crop meant that high incidences of CBSD in surrounding fields had little impact on spread into the experimental plots. These results suggest that farmers wishing to plant susceptible cassava cultivars or those growing cassava for commercial seed production could do so during Masika to reduce the effects of degeneration. However, for breeders, testing new clones/varieties during Vuli when maximum effects of vector abundance and disease pressure are realized would be more beneficial. The significant differences in *B. tabaci* abundance among varieties indicated an important research area that can be explored to strengthen the efforts to combat CBSD.

Varieties differed greatly in CBSD infection. CBSD shoot symptoms showed a marked increase through successive planting cycles in Masika for the susceptible variety (Kikombe) as well as for the tolerant variety Kiroba. Whereas the tolerant Kiroba (Maruthi et al. 2014; Mohammed et al. 2015) showed mild to severe shoot symptoms throughout Masika, it was relatively easy to select sufficient symptom-free planting material for the new Masika crop cycle. The performance of Kiroba was markedly different during the third cycle in Vuli, when the variety developed very severe symptoms similar to the susceptible variety Kikombe. For Kipusa, which also had shoot symptoms, no significant changes were observed between successive planting cycles in either Masika or Vuli seasons. The absence of shoot symptoms in most of the elite varieties used in this study suggests that tolerant varieties can remain productive over several planting-replanting cycles (at least four cycles in Masika and at least 3 years in Vuli), allowing farmers ample time to realize economic benefits of their crop before sourcing new stocks of virus-free planting material. The lower incidence (1.5%) of CBSD in initially virustested material sourced from clean stocks compared with recycled material (7.0%) highlights the value of managing CBSD through the production of certified cassava planting material, which incorporates a virus-testing stage (Kanju et al. 2003).

An important epidemiological aspect was noted in the proportions of virus types detected over the duration of this study. The number of virus tests (>1,000 samples) reported in this study is probably the largest ever reported from this part of Africa. Virus infection was correlated with CBSD incidences, where UCBSV was the most predominant species (>80%), suggesting that it was the virus species that was most associated with degeneration in the affected varieties. The fact that this study reports UCBSV as the most frequent species is in contrast with a study in Uganda, where CBSV was reportedly the most frequent virus in field-grown cassava (Ogwok et al. 2015). Furthermore, in our study, virus species frequencies were similar between the Masika and Vuli seasons. Proportions of UCBSV reported in this study were similar between the susceptible variety Kikombe and the tolerant Kiroba, which means that, whereas a given virus species might elicit similar host response in the same plant part of different varieties, these effects may not be similar in all of the plant parts for both varieties. In this study, Kikombe was most severely affected in both shoot and root components, whereas Kiroba was most affected in the foliar component. Whereas in this study, UCBSV was seemingly the most virulent, some previous reports recorded CBSV as the most virulent (Kaweesi et al. 2014; Mohammed et al. 2012; Winter et al. 2010). These results highlight that the distribution and frequency of CBSIs are determined by a combination of several factors rather than just the environment where the host is located. Studies have suggested that current CBSIs are relatively more virulent than those that existed previously (Alicai et al. 2016, Kawuki et al. 2017).

The cassava varieties tested in this study responded differently to CBSI infections, thus resulting in different patterns of degeneration. One pattern (strong degeneration), demonstrated by Kikombe involved the development of increasing incidences of severe CBSD symptoms over repeated planting cycles. The second pattern (moderate), shown by Kiroba, comprised increasing incidences of severe shoot symptoms over time but low incidences of severe root symptoms throughout. The third pattern (mild; Kipusa) was characterized by very severe shoot and root symptoms at the outset that decreased to mild symptoms by the end of the study period. The fourth pattern (delayed; Chereko, KBH2002-135, Kizimbani, and Mkuranga1) was characterized by the absence of shoot symptoms but the development of mild root symptoms over the course of the experiment. This last group consisted of varieties for which there is a “delay” in expressing symptoms when recycled several times, although virus testing revealed that all were infected by CBSIs at some stage.

Significantly different CBSD incidences were recorded between the Vuli and Masika experiments reported in this study, showing a strong association between shoot and root symptoms. Higher incidences in shoot and root symptoms were recorded in Vuli than in Masika, and these were associated with higher levels of degeneration in Vuli. Varieties that had the most severe CBSD shoot symptoms also had the most severe root degeneration and vice versa. Kikombe had the highest CBSD shoot incidence overall, and it also had the most severe damage recorded for its roots. Kiroba and Kipusa had moderate to severe shoot symptoms and overall moderate root symptoms. Degeneration was noted with respect to both shoot and root symptoms. The pattern of CBSD incidences observed in this study was similar to that reported by Ndyetabula et al. (2016) (i.e., plant total incidence was highest, and unusable root incidence was the least). It was noted in this study, however, that root incidences were generally higher than those of shoot incidences overall.

Results of this study showed that CBSD reduced marketable yield. Tuberous root yield, shoot yield, root HI, and marketable yield were all higher in the Masika season than in Vuli. Differences in planting date can give rise to important differences in the epidemiology of insect-vectored virus diseases (Adipala et al. 1998). Planting in Masika resulted in fewer vectors, less virus, and higher marketable yield. However, planting in Vuli resulted in higher vector abundance, more viruses, and lower marketable yield. In this study, the highest CBSD incidences were observed in Vuli, where there was also lower marketable yield. Impacts of higher virus inoculum pressure can result in significant yield losses within just one planting cycle, especially when susceptible varieties are used (Adikini et al. 2015; Mwanga et al. 2007). There are socioeconomic reasons why farmers in coastal Tanzania favor planting cassava in the Vuli season. For example, maize is the preferred crop in Masika, because this will not grow well when planted in the Vuli season. This is compounded by labor constraints, which are greater at the start of the Masika season than in Vuli. Where a grower’s primary focus is on cassava, however, the evidence presented in this study demonstrates the great advantage of planting cassava in the main rains received during the Masika season.

Moreover, our results show that degeneration resulted in yield reduction over the course of the experiment. CBSD-affected varieties showed a progressive decline in shoot and root yield over the course of the experiment. New material was always least affected by CBSD, except for the susceptible variety Kikombe in Vuli. Kikombe showed the greatest yield reductions over time compared with the less susceptible variety Kipusa and the tolerant variety Kiroba. Reduction in yield meant that there was a significant decrease in marketable yield for Kikombe over the course of the Masika and Vuli experiments. The other varieties were not affected by CBSD and had no apparent yield reduction over time associated with CBSD. Importantly, yield reductions became greater over time. Although yields of CBSD-affected varieties were similar in both Masika and Vuli at the outset, by the end of the experiment, degeneration meant that Vuli yields in affected varieties were significantly lower than Masika yields for the same varieties. Although no differences were recorded for DM between Masika and Vuli, the differences observed between new (high; 34.1%) and recycled (low; 33.2%) materials suggested that the effects of degeneration significantly compromised the overall quality of the tuberous root harvest, because recycled material was the most affected by CBSD. Whereas degeneration owing to CBSD has the greatest effects on crop yield, other factors associated with recycling planting material might also contribute to the overall performance of a crop, and it would be worth examining these more thoroughly in future experiments. Degeneration did not affect HI. The results of this degeneration study on elite cassava varieties planted in a bimodal rainfall site in coastal Tanzania showed significant reductions over time in root and shoot yields concomitant with increases in CBSD incidence. Although faster degeneration rates were demonstrated in the short rains season Vuli, slower degeneration rates (accompanied by lower CBSD incidences and severities) and higher yields were recorded in the long rains season Masika. Tolerant and/or resistant varieties were less affected by degeneration, having little to no shoot symptoms for some of the varieties even after recycling for 3 to 4 years, but significant root necrosis symptoms were observed for most of the varieties. Similarly, all elite varieties were infected by CBSIs, although they had lower incidences than the susceptible variety (Kikombe). This study clearly demonstrates the yield benefits of using new planting material for each planting cycle, therefore encouraging farmers in the study area and elsewhere where cassava is important in sub-Saharan Africa to use virus-free planting material to overcome effects of virus-associated degeneration (Thomas-Sharma et al. 2016). Because CBSD continues to spread westward through Central Africa and toward the western part of the continent, it will become increasingly important to raise farmer awareness about the benefits of virus-free planting material in all affected regions as well as those that are imminently threatened.

This study further showed that, whereas tolerant/resistant varieties can be recycled for at least 3 years in the Vuli season, farmerpreferred varieties can only be planted over successive cycles during the low-CBSD pressure season Masika. Additional research over a wider geographical area should shed more light on the effects of degeneration on recycling planting material. Findings of this study further suggest that breeders should test new varieties during the Vuli season to capture the greatest effects of CBSD pressure in coastal Tanzania. To the best of our knowledge, this report presents the first study on degeneration owing to cassava viruses. It seems that degeneration might not just be owing to cassava viruses, because there may be other contributing factors. A study from 2015 on degeneration of a root crop for East Africa was on sweet potato (Adikini et al. 2015). As in our study on cassava, this showed that, although there were high levels of degeneration in susceptible varieties, for resistant varieties, the decline in performance over several cycles was not sufficiently important to make it worthwhile for farmers to purchase new planting material each year. Although many farmers still grow for subsistence, there is an increasing trend in East Africa and elsewhere toward the commercialization of the cassava crop. We anticipate that the results presented here will be of great value in guiding commercial producers toward the best choice of planting season and variety when designing new cassava enterprises.
